# Privacy Barriers in Health Monitoring: Scoping Review

**DOI:** 10.2196/53592

**Published:** 2024-05-09

**Authors:** Luyi Sun, Bian Yang, Els Kindt, Jingyi Chu

**Affiliations:** 1 Department of Information Security and Communication Technology Faculty of Information Technology and Electrical Engineering Norwegian University of Science and Technology Gjøvik Norway; 2 Centre for IT & IP Law Faculty of Law and Criminology KU Leuven Leuven Belgium; 3 Administrative Law Faculty of Law China University of Political Science and Law Beijing China

**Keywords:** privacy attitudes, health monitoring technologies, privacy concerns, privacy barriers, legal concerns, social psychology

## Abstract

**Background:**

Health monitoring technologies help patients and older adults live better and stay longer in their own homes. However, there are many factors influencing their adoption of these technologies. Privacy is one of them.

**Objective:**

The aim of this study was to provide an overview of the privacy barriers in health monitoring from current research, analyze the factors that influence patients to adopt assisted living technologies, provide a social psychological explanation, and propose suggestions for mitigating these barriers in future research.

**Methods:**

A scoping review was conducted, and web-based literature databases were searched for published studies to explore the available research on privacy barriers in a health monitoring environment.

**Results:**

In total, 65 articles met the inclusion criteria and were selected and analyzed. Contradictory findings and results were found in some of the included articles. We analyzed the contradictory findings and provided possible explanations for current barriers, such as demographic differences, information asymmetry, researchers’ conceptual confusion, inducible experiment design and its psychological impacts on participants, researchers’ confirmation bias, and a lack of distinction among different user roles. We found that few exploratory studies have been conducted so far to collect privacy-related legal norms in a health monitoring environment. Four research questions related to privacy barriers were raised, and an attempt was made to provide answers.

**Conclusions:**

This review highlights the problems of some research, summarizes patients’ privacy concerns and legal concerns from the studies conducted, and lists the factors that should be considered when gathering and analyzing people’s privacy attitudes.

## Introduction

### Background

The proportion of older adults around the world is growing sharply. According to the 2021 aging report published by the European Commission [1], the ratio in the European Union (EU) between people aged ≥65 years and those aged 20 to 64 years (also known as the demographic old-age dependency ratio) will increase greatly in the coming decades, from approximately 34% in 2019 to 59% in 2070. In Norway, the population of older adults (aged ≥65 years) will increase from 17.4% in 2019 to 27.8% in 2070 [1]. Because of this, the term “aging in place” was put forward in social policy, which refers to providing assisted living facilities to enable older adults to remain in their own homes for as long as possible [2]. Various ongoing research projects in ambient assisted living technologies are being, or have been, conducted to help older adults, such as the European AALIANCE2 project; the Ambient Assisted Living Joint Programme, financed by the European Commission [3]; and the Active and Assisted Living Programme, also financed by the European Commission [4].

In the meantime, patients are also in need of health monitoring systems. The aging population and patients’ growing needs for health care support have facilitated the development of different types of health monitoring and assisted living technologies, such as socially assistive robots, wearable trackers, telemedicine, image sensors, and so on. According to the study by Rostad and Stokke [5], these technologies can be categorized into localization technologies (eg, GPS), compensation technologies (eg, remote control of light and heating, robot vacuums, and cognitive or physical aids), safety technologies (eg, social alarm systems and fall detection sensors), technologies for social contact (eg, tablet computers, smartphones, and gaming devices), therapeutic robots, and treatment technologies (eg, medical remote monitoring and automated pill dispensers), all used for different purposes in health monitoring.

The application of the aforementioned intelligent devices is supposed to enhance the quality of patients’ lives. Nevertheless, various factors impact patients’ acceptance of health monitoring devices [6], including intervention factors (eg, privacy concerns, security concerns, frequency, a lack of awareness, and the observability of outcomes), environmental factors (eg, social influence, social implication, change in technology use by society over time, and group participation), technology factors (eg, complexity, reliability, interface design, compatibility, functionalities, features, aesthetics, and cost), individual factors (eg, level of innovativeness, level of technology learnability, and living alone), psychological factors (eg, attitudinal factors and perception factors), support and training factors (eg, training, quality of training, and facilitating support) in general. Among all these factors, our attention was seized by *privacy concerns*.

There are different types of privacy; therefore, privacy concerns can be analyzed from different perspectives. The way that researchers distinguish privacy has reflected their different concerns arising from their professional backgrounds; for example, Rosenberg [7] distinguished 3 kinds of privacy: territorial privacy, individual privacy, and informational privacy. Clarke [8] outlined 4 types of privacy: privacy of a person, privacy of personal data, privacy of personal behavior, and privacy of personal communication. However, with the development of emerging technologies, different categories arose. Some researchers extended the categories formulated by Rosenberg [7] and added 3 more categories: privacy of thoughts and feelings, privacy of location and space, privacy of association (including group privacy) [9]. More specifically, in different scenarios, users have different privacy concerns, and these concerns can be categorized case by case; for instance, web-based social network users can have 4 dimensions of privacy concerns: virtual territorial privacy, factual privacy, interactional privacy, and psychological privacy [10]. Furthermore, Serenko [11] narrowed the scope in health care and put forward 3 privacy dimensions: informational privacy, physical privacy, and psychological privacy. These 3 privacy dimensions were regarded as determinants of patient behavior in health care.

Although privacy concerns are known to be barriers for patients with regard to adopting health monitoring technologies according to some studies [12], it is not sufficient to analyze factors impacting the adoption of health monitoring technologies individually because these influential factors may impact each other; for example, despite the fact that privacy concerns are included in intervention factors [6], psychological factors, as well as support and training factors with regard to privacy awareness, can also impact patients’ privacy concerns. Patients’ awareness of privacy-related laws in the health care environment will also influence their privacy concerns and decision-making out of respect for authority [13]. Considering privacy issues in society, privacy attitudes and concerns are always analyzed from the legal perspective. For patients, their privacy attitudes may have a straight impact on the informed consent process, and they are expected to know how to obtain legal aid in case they encounter technology abuse, or their privacy is intruded upon. For technology vendors, industry standards or privacy policies need to be carefully checked and complied with before their product is released. They need to carry out data protection impact assessments to minimize privacy risks [14]. Apart from older patients who are most in need of health monitoring and assisted living technologies, members of the general public are also potential users of these technologies as they age or develop health conditions. Furthermore, with regard to members of the general public, their prevalent uncertainty regarding, and trust issues with, technologies may prompt legislators to adopt a more cautious and conservative approach with regard to such technologies. However, *privacy-friendly* approaches can be seen as a way to motivate technology vendors to be more transparent and, on the one hand, foster *privacy by design*, while, on the other hand, promote social awareness and trust by bridging the information gap. In all, various factors and their relationships need to be always taken into consideration at the same time. Meanwhile, the question persists as to whether privacy concerns are truly barriers because of the rapid changes in society, such as the development of privacy-enhancing technologies. Thus, the rationality of privacy concerns should also be discussed.

In the past few years, researchers have conducted a series of studies to gather different privacy attitudes or privacy concerns regarding health monitoring and assisted living technologies from people with different demographic characteristics. However, the results vary from study to study not only because of the different user groups to which the participants belong (eg, older adults’ attitudes compared to those of younger adults and female participants’ attitudes compared to those of male participants) but also because of the different experimental approaches used and the different scenarios provided in these studies.

### Research Questions

In summary, the implementation of assisted living technologies in the aging population era faces several challenges. First, multiple factors impede patients’ adoption of these technologies, and the interrelations among these factors have not been thoroughly explored. Second, the extent to which privacy concerns affect technology adoption needs further investigation. Third, the study design and current results require consolidation for researchers to make meaningful improvements. Addressing these challenges, we pose 4 research questions and conduct a scoping review with the aim of providing an overview of the privacy barriers in health monitoring from current research and elucidating answers to these research questions. The four research questions are listed herein, and the answers to these questions are provided in the Results section.

1. What are the influential factors that lead to different privacy attitudes and concerns?
2. How will the methodologies used in different studies influence participants’ privacy awareness with regard to health monitoring technologies from the perspective of social psychology?
3. What are the legal challenges regarding people’s privacy attitudes and concerns today?
4. What should be taken into consideration in subsequent studies related to privacy attitudes and concerns in the context of social psychology?

On the basis of the selected articles, we have summarized 5 hypotheses particularly related to the second challenge, which are clarified in the Results section. The contribution of this paper includes observing the inconsistency of these hypotheses, looking into experimental approaches in each article, and seeking answers to the 4 research questions. We have tried to come up with suggestions that should be taken into consideration comprehensively before implementing health monitoring technologies.

## Methods

### Overview

A scoping review was conducted to explore the privacy attitudes of different groups of participants in the context of legal norms and social psychology in health monitoring technologies by adopting the PRISMA (Preferred Reporting Items for Systematic Reviews and Meta-Analyses) statement (Multimedia Appendix 1) [15]. Scoping reviews include all quantitative, qualitative, and mixed methods studies that are identified as literature on a particular topic or research area [16,17]. They differ from systematic reviews but can be used to inform systematic reviews because more specific questions are usually addressed in a more precise systematic review [18]. Of note, there are other approaches to evidence synthesis for systematic reviews, such as realist reviews [19], mixed methods reviews [19], concept analyses [20], and so on. In this study, with the aim of identifying and mapping the available studies, examining how research is conducted in a certain field, summarizing findings, and analyzing results, a scoping review is the best choice compared to other approaches.

### Eligibility Criteria

We conducted a review for articles published between January 1, 2016, and March 31, 2022. Search parameters were established to identify articles published during this period regarding different participants’ privacy attitudes with regard to health monitoring technologies as well as legal norms regarding privacy in health monitoring in Norway, the EU, and the United States. For an in-depth investigation into the research questions, we acknowledge the regional characteristics evident in previous studies, often shaped by factors such as cultural backgrounds and legal norms. To address this, we have selected these regions. Specifically, this decision is motivated by 2 key considerations. First, EU policies extend to Norway, the authors’ country of residence, thereby potentially impacting health service delivery and the deployment of assisted living technologies. Second, certain EU countries share a common cultural background, suggesting that individuals in these regions may harbor more similar privacy perspectives than individuals in other locations. Furthermore, studies published in the United States were included because it is one of the most developed countries owning quantities of health monitoring technologies. It is worth mentioning that for the articles we identified, even if the authors did not specify the review region or if the authors’ countries of residence were outside the region, we still included these articles because they provided comprehensive views. Other than region specification, studies were included if they (1) reflected the privacy attitudes or privacy concerns of any group of people, (2) reflected any legal concerns or legal frameworks that should be taken into account, (3) were peer-reviewed publications, and (4) were written in English. All study methods (quantitative, qualitative, and multimethod) were eligible for the review.

### Search Terms, Strategy, and Sources

Instead of searching for privacy barriers directly, we sought studies relevant to people’s privacy attitudes or legal norms regarding privacy in the health monitoring environment and tried to summarize the barriers described in these studies. The literature search was conducted by listing the following search terms in the search string: (“privacy attitudes” OR “legal norms”) AND ((“healthcare monitoring” AND “nursing homes”) OR “homecare monitoring”) AND (“Norway” OR “EU” OR “the U.S.”). The sources of the articles on privacy attitudes and legal norms were mainly 5 databases: Semantic Scholar, PubMed, IEEE Xplore, ScienceDirect, and Scopus. As no relevant articles met the eligibility criteria in IEEE Xplore and ScienceDirect, only articles in the rest of the 3 databases were included. Additional works identified in other databases, such as ACM Digital Library, were categorized into *other sources* because we sorted the articles by relevance and scanned the results directly based on the title and abstract provided at the first attempt instead of following the PRISMA steps strictly, which was the approach we followed for the 5 main databases. Therefore, instead of making an exhaustive selection, we merely added the most relevant and important works.

### Study Selection

**Figure 1:**
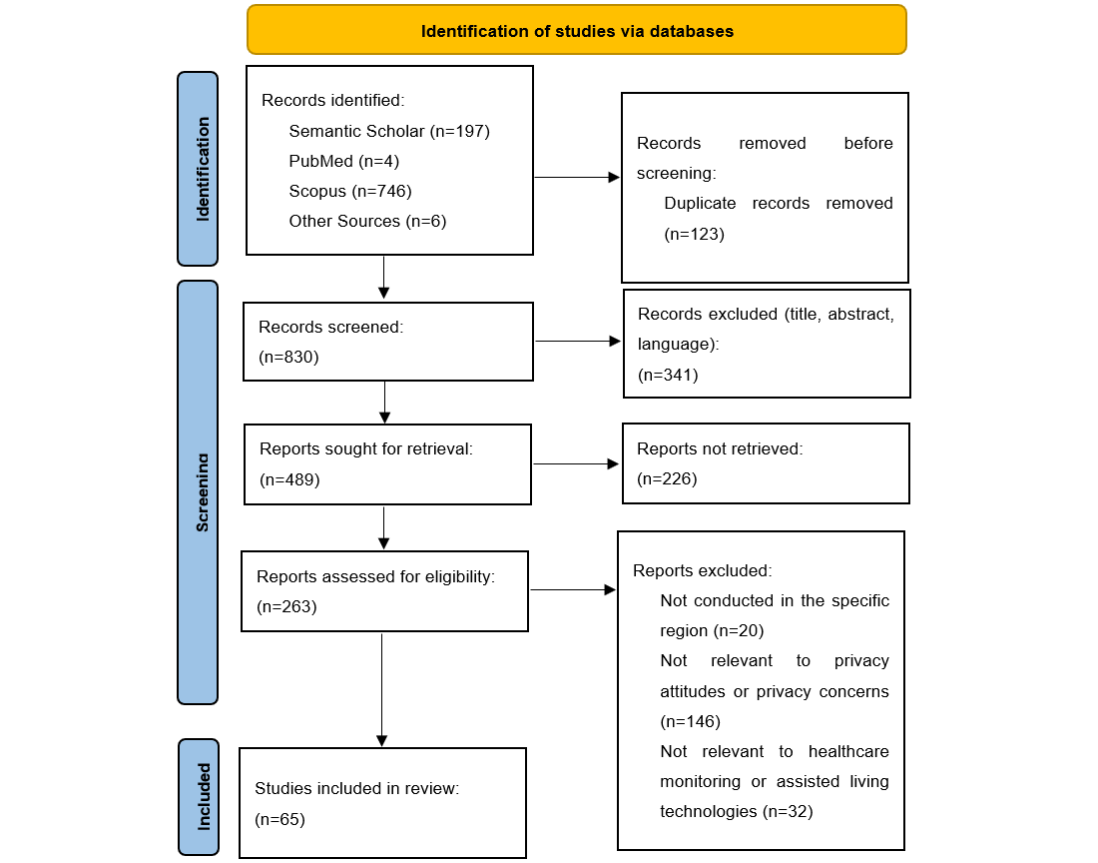
The review steps.

The PRISMA flow diagram is presented in Figure 1. The search process resulted in the identification of 953 studies. Before the screening, 122 (12.9%) duplicate records were removed from these 953 studies. The first screening was performed on the title, abstract, and language, and 341 (41%) of the 830 articles were identified as not meeting the eligibility criteria. Of the remaining 489 articles, 226 (46.3%) could not be retrieved, leaving 263 (53.7%) reports for assessment. After the second screening, of the 263 articles, we excluded 198 (75.2%) because they (1) were not conducted in the regions specified, (2) were not relevant to privacy attitudes or privacy concerns, and (3) were not relevant to health monitoring or assisted living technologies, leaving 66 (24.8%) articles for the final review, from which we extracted and categorized useful information.

### Data Extraction and Categorization

The methods for data extraction and categorization were established through the literature review process. Useful information was extracted and input into a form, including title; authors; year of publication; region; topic; technology; participant inclusion criteria (if any); study design; location of the study; key findings; and laws, rules, regulations, directives, and policies mentioned.

### Categories

The categories we formulated are presented in Textbox 1.

**Categorization of the articles included for review.**

**Categories**

- Article information: title, authors, year of publication, and region
- Topic: identified and categorized based on the field covered by the articles; instead of setting the topic as “privacy attitudes” or “legal norms” in general, 5 topics were defined (privacy attitudes, privacy concerns, legal concerns, legal frameworks, and privacy barriers); some of the articles have covered several topics at the same time; reasons for classification are clarified in the *Results* section
- Technology: includes health monitoring technologies mentioned in the article; some of the articles have covered a specific device (eg, human behavior modeling [21]), while some provide privacy attitudes or legal norms of a general designation, such as smart home technology
- Participant inclusion criteria: specifically created for studies with participants invited to take part; inclusion criteria include the number of participants, age, nationality, whether they have any diseases, and place of residence
- Key findings: any information related to the 5 topics listed in the Topic category
- Laws, rules, regulations, directives, and policies mentioned: specifically created for studies covering legal frameworks or legal barriers; all legal documents mentioned in the articles were extracted

## Results

### Overview

In this section, we will provide the results in the form of categories. As mentioned in the Eligibility Criteria subsection, the review was focused on Norway, the EU, and the United States, or reviews worldwide. When it comes to review articles, they are included regardless of region. Among the 65 articles, there were 4 (6%) from France, 2 (3%) from Finland, 1 (2%) from Sweden, 3 (5%) from Germany, 1 (2%) from Ireland, 2 (3%) from Italy, 3 (5%) from the Netherlands, 5 (8%) from Norway, 2 (3%) from Poland, 1 (2%) from Portugal, 1 (2%) from Spain, 1 (2%) conducted jointly in Germany and Denmark, and 30 (46%) from the United States. Of the remaining 9 articles, 1 (11%) discussed telemonitoring at the EU level; 1 (11%) reviewed laws, standards, and recommendations applicable at the EU level; and 7 (78%) were literature reviews whose authors’ countries of residence were not part of the specified regions. As the results of the reviews were general in nature, they were not categorized into specific areas.

### Privacy Attitudes, Privacy Concerns, and Privacy Barriers

Articles reflecting privacy concerns were classified into 3 subcategories: privacy attitudes, privacy concerns, and privacy barriers. According to Kokolakis [22], although privacy attitudes and privacy concerns have a close relationship, they differ from each other because privacy attitudes are bound to specific contexts such as the appraisal of specific privacy behaviors, while privacy concerns are not, and they can be generic. It is worth mentioning that the articles that either gathered participants’ general privacy concerns or participants’ privacy attitudes were included in the review. The key findings extracted from the results should be categorized as privacy concerns according to the definition. However, we set the topic as *privacy attitudes* if any privacy attitudes were gathered in these studies. Thus, articles covering either privacy concerns or privacy attitudes were included when we compared the findings.

In contrast to the studies that gathered and analyzed participants’ privacy attitudes or concerns, 11 (17%) of the 66 studies [23-33] discussed people’s adoption of health monitoring technologies. Although some of these studies, such as the study by Charness et al [26], also recruited participants, gathered their privacy attitudes, and analyzed results from the attitudes (similar to the methods used by Sánchez et al [21] and Caldeira et al [34]), these studies provide a broad view from the perspective of technology adoption and acceptance; for instance, in the study by Biermann et al [25], researchers came up with several barriers to technology adoption, such as financial reasons, restriction of privacy, and a feeling of surveillance. Among all barriers, concern regarding privacy is merely one of the factors that may influence participants’ technology adoption. Therefore, even if the findings reflecting privacy concerns are similar to those reflecting privacy attitudes and privacy barriers, articles stating that concern regarding privacy is one of the barriers to the adoption and acceptance of assisted living technologies were classified into a different category: privacy barriers.

### Contradictions Among Perceptions of Privacy in Key Findings

All included studies reflect privacy attitudes, privacy concerns, and privacy barriers, which show many contradictions. In general, the results can be classified into five hypotheses according to the extent of participants’ privacy concerns: (1) participants do not have privacy concerns (Table 1); (2) participants have privacy concerns, and they are a major barrier (Table 2); (3) participants have privacy concerns, but they do not seem to be a significant barrier (Table 3); (4) participants’ privacy concerns vary from person to person, and there is insufficient statistical evidence across a large population to validate whether the concerns will have a significant influence (Textbox 2); and (5) participants have privacy concerns, and their perceptions of privacy are influenced by their background (Table 4). It is worth mentioning that some of the studies satisfied 2 hypotheses at the same time because the fifth hypothesis does not violate the second, third, or fourth hypothesis.

**Table 1:** Studies that provide evidence for the first hypothesis.

Study	Methodology	Evidence
Sánchez et al [21]	Interview (exploratory qualitative approach)	“It was noteworthy that the majority of participants had no privacy concerns.”
Caldeira et al [34]	Interview (qualitative approach)	“Privacy did not seem to be a significant issue for our informants.”

**Table 2:** Studies that provide evidence for the second hypothesis.

Study	Methodology	Evidence
Schomakers et al [35]	Qualitative prestudy+quantitative main study (multimethod approach)	“As an important barrier, privacy requirements should thus be considered for mHealth [mobile health] apps for aftercare.”
Vassli and Farshchian [23]	Systematic review	“Many studies found that some or all participants have concerns about privacy. Privacy is regarded as a ‘bigger barrier to adoption, more so than usability.’”
Harrington et al [36]	Questionnaire (quantitative study)	“Privacy was among the leading concerns regarding SARs [socially assistive robots] among the current sample of older Americans.”
Choi et al [37]	Questionnaire+semistructured interview (multimethod approach)	“The participant feedback suggests that perceived privacy concerns, perceived usefulness, and curiosity to technology were strong factors when considering which device to have installed in their home.”
Tural et al [38]	Web-based and in-person surveys+focus group (multimethod approach)	“Privacy and security of personal information seem to be a core issue for willingness to use smart home products as also highlighted by others.”
Attié et al [39]	Survey	“Privacy concerns are the main obstacles to the adoption of SCOs [smart connected objects].”
Lederman et al [31]	Review	“Other researchers suggested that risk perception that is influenced by concern over privacy, security and the learning-curve can have a negative impact on the adoption of IoT [Internet of Things] solutions by medical staff...These risks to privacy and security are a major challenge for IoT in healthcare.”
Karlsen et al [40]	Review	“The lack of security and privacy was a prominent concern due to the constant recording of data and location tracking that comes with the use of a smartwatch.”
Gimpel et al [41]	Survey	“In healthcare digitalization, privacy concerns are one of the major barriers for individuals to accept and use healthcare technologies.”
Mujirishvili et al [42]	Scoping review	“With privacy being a major barrier to video-based AAL [active and assisted living] technologies, security and medical safety were identified as the major benefits across the studies.”
Wilczewski et al [43]	Questionnaire	“Participants commented on privacy concerns with providing personal information to the chatbot. This category had the majority of negative comments (n=10/15 codes; 66.7%) with some participants finding the chatbot ‘a bit intrusive.’”

**Table 3:** Studies that provide evidence for the third hypothesis.

Study	Methodology	Evidence
Jaschinski et al [44]	Web-based survey (qualitative approach)	“Older adults’ privacy concerns were secondary to the perceived benefits of AAL [Ambient Assisted Living] in terms of health, safety and independence.”
Gettel et al [28]	Scoping review	“One study highlighted that older adults were concerned about privacy, but other studies found that privacy was not a barrier to AAL [Ambient Assisted Living] technology adoption.”
Chung et al [45]	Survey (qualitative approach)	“The proportion for the privacy concern increased slightly, though not statistically significant, indicating that participants were not bothered by the existence of the device at home.”
Fruchter and Liccardi [46]	Web-based review	“While we found that privacy and security related issues are present within our corpus, our results suggest these topics related to home assistants are rarely voiced, or openly reported by consumers in their online reviews. We can conclude that, for the most part, consumers who review home assistants tend to not discuss privacy or security concerns.”
Piau et al [47]	Web-based survey (qualitative approach)	“Less than a third were concerned about privacy breaches when using these technologies.”
Tan et al [48]	Semistructured interviews (qualitative approach)	“When asked about potential privacy or security concerns, our analysis of participant responses surfaced 6 types of reactions that may explain why they expressed little concern with privacy and security.”
Schomakers and Ziefle [49]	Questionnaire (quantitative approach)	“Our data suggests that privacy concerns are outweighed by security-related benefits in the acceptance decisions, as long as certain lines are not crossed—the unacceptable and must-have characteristics.”

**Studies that provide evidence for the fourth hypothesis.**

**Study and methodology**

- Randall et al [50]: focus group or qualitative approach
- Gerłowska et al [51]: literature review
- Soro et al [52]: review
- Wang et al [53]: focus group+survey (multimethod approach)
- Pilozzi and Huang [54]: no methods directly related to the results
- Pekmezaris et al [55]: focus group+interview (multimethod approach)
- Biermann et al [25]: web-based questionnaire (empirical quantitative approach)
- Kodate et al [56]: questionnaire (quantitative approach)
- Berridge et al [57]: survey (qualitative approach)
- Mittelstadt [58]: systematic survey
- Koo and Fallon [59]: interview (qualitative approach)
- Joe et al [60]: focus group+questionnaire (multimethod approach)
- Chan et al [27]: literature review
- Chan et al [29]: literature review
- Sánchez et al [61]: literature review
- Hjelm et al [62]: semistructured interview (qualitative approach)
- Cristiano et al [63]: interview+focus group (qualitative approach)
- Zhang et al [64]: questionnaire (quantitative approach)
- Mallinson and Shafi [32]: review
- Guazzini et al [65]: questionnaire+focus group (multimethod approach)
- Wan et al [66]: literature review+semistructured interviews (multimethod approach)
- Zheng et al [67]: semistructured interviews (qualitative approach)
- Yao et al [68]: focus group+co-design activities (qualitative approach)
- Ahmad et al [69]: semistructured interviews (qualitative approach)
- Kheirinejad et al [70]: questionnaire (quantitative approach)

**Table 4:** Studies that provide evidence for the fifth hypothesis.

Study	Methodology	Evidence	Influential factors
Schomakers et al [35]	Qualitative prestudy+quantitative main study (multimethod approach)	“Acceptance and privacy depend on the context and type of the technology.”	Context and type of technology
Vassli and Farshchian [23]	Systematic review	“Systems that are considered intrusive or causing infringement on privacy might still be accepted by older adults if their health needs are great enough.”	Age and health needs
Shin et al [24]	Systematic review	“Personal trust and the device’s usability could affect users’ privacy perception of wearable activity trackers.”	Personal trust and the device’s usability
Soro et al [52]	Review	“When it comes to privacy, older adults are very thoughtful and want to be empowered and to retain the sense of the home as a haven with respect for their autonomy.”	Age and autonomy
Wang et al [53]	Focus group+survey (multimethod approach)	“Older adults scored lower in the privacy pragmatic and unconcerned categories and much higher in the privacy fundamentalist category.”	Age
Reeder et al [71]	Semistructured interview (qualitative approach)	“Older women’s privacy concerns related to sensor technology can vary according to their sociocultural context (e.g., Korean American older adults and Korean older adults VS Caucasian older adults).”	Age, gender, and sociocultural context
Pilozzi and Huang [54]	No methods directly related to the results	“Individuals with Parkinson’s disease were almost three times more likely to have data-privacy related concerns than controls.”	Disease (Parkinson disease)
Halvorsrud et al [72]	Interview (qualitative approach)	“This study reveals that older adults’ perspectives on assistive technology (AT) are multifaceted and complex, and can partly be explained by the interacting factors in the HAAT [human activity assistive technology] model: person, technology, environment, and context.”	Person, technology, environment, and context
Langer et al [73]	No methods directly related to the results	“Women tend to be more concerned with privacy and safety than men, often preferring enclosed latrines in or near their homes.”	Gender
Jaschinski and Ben Allouch [74]	Semistructured interview (qualitative approach)	“Informal caregivers had a more positive attitude than care receivers.”	User role
Charness et al [26]	Questionnaire (quantitative approach)	“Older adults, particularly males, showed less concern than younger adults about privacy.”	Age
Chan et al [27]	Literature review	“There is tension between assistance and autonomy, or privacy and independence that characterizes the individual’s judgment in using telehealth technology.”	Assistance, autonomy, and independence
Sánchez et al [61]	Literature review	“Privacy can be compromised for persons in need of support...People with higher risk of harm often require intense surveillance to avoid unsafe situations.”	Support and safety
Łukasik et al [75]	Questionnaire (quantitative approach)	“Medical students were more aware of privacy issues in the statement concerning the possibility of switching off the robot in specific situations.”	User role
Lanne and Leikas [30]	Semistructured interview+literature review (multimethod approach)	“Using AI [artificial intelligence] in social and health care contains many general challenges. Some of the most commonly discussed topics were related to social trust and the experience of autonomy, power structures, privacy concerns, transparency, and biases leading to unfair treatment of individuals and patient groups.”	Autonomy, trust, and transparency
Simpson et al [76]	Review	“Privacy concerns are reported as being the main reason patients may choose not to share data in a clinical context, though these concerns mostly relate to the potential for future sharing with external third parties.”	Potential use of the data
Zhang et al [64]	Questionnaire (quantitative approach)	“Privacy awareness (*P*=.08) has positive effects on privacy concerns.”	Privacy awareness
Seberger and Patil [77]	Semistructured interviews (qualitative approach)	“In the context of pandemic mitigation technology, including app-based tracking, people perceive a core trade-off between public health and personal privacy.”	Public health
Kolakowsk et al [33]	Literature review	“Cultural barriers will likely result in unequal diffusion of robot use in elderly assistance over time.”	Social context
Chaparro et al [78]	Review	“There is a list of factors that affect the attitude and intention to use technologies supporting independent living. These personal and device-related factors comprise user expectancy, biophysical ageing restrictions, anxiety, the previous required knowledge, intrinsic motivation, personality and privacy concerns.”	Emotion, knowledge, and personality
Gimpel et al [41]	Survey	“Several studies have shown that Germans have higher privacy concerns than citizens in most other countries. Most authors attribute this to German’s historical legacy.”	Region and sociocultural context
Zheng et al [67]	Semistructured interviews (qualitative approach)	“IoT [Internet of Things] device users in different regions may have differing privacy concerns. For example, American users may be generally more accepting of data collection by industry versus the state, in contrast to consumers in Europe...Since interview participants expressed greater privacy concern about devices that record voice and video, we recommend that such visual indicators be used extensively to indicate these activities, especially in devices traditionally without recording capabilities (e.g. doorbells, lightbulbs, etc.).”	Region and data type collected by devices
Yao et al [68]	Focus group+co-design activities or qualitative approach	“In general, bystanders had more privacy concerns in the temporary residence scenario and the playdate scenario than the cohabitant scenario. Bystanders also expressed more concerns regarding the video and audio data collected by devices with microphones and cameras (e.g., voice assistants, security cameras) but barely any concern with other devices (e.g., smart coffee makers).”	User role and residence scenario
Ahmad et al [79]	Semistructured interviews	“Older participants may have different privacy concerns as well as different interpretations of IoT [Internet of Things] designs and indicators. Although one worry may be that younger populations are less concerned about their privacy, we note that Singh et al. [reference citation] found that when it comes to sharing information with smart devices, younger adults are more reluctant than older adults.”	Age

## Discussion

The aforementioned contradictory hypotheses have led to the formulation of our research questions, which we attempt to answer in this section.

### Research Question 1: What Are the Influential Factors That Lead to Different Hypotheses?

To identify the influential factors, we looked into the methodologies used in these studies in detail. To sum up, qualitative, quantitative, and multimethod studies as well as reviews were included in these studies. Except for reviews, the other 3 approaches recruited participants during the study. On the basis of the participant inclusion criteria, we found that the number of participants would influence the results. For those studies that concluded that the majority of participants do not have privacy concerns, the number of participants recruited was small [21,34]. Hence, one could argue that there might have been sampling bias in the qualitative approach applied by the studies conducted. As the number of participants increased, the fact that people had privacy concerns seemed to become a common conclusion.

Nevertheless, it still seemed hard for researchers to come to an agreement on the importance of privacy issues. Some stated that privacy is an important barrier without verification and regarded it as a consensus [35]. However, according to a scoping review of ambient assisted living technology adoption, most studies found that privacy was not a barrier [28]. Because of the uncertainty mentioned above [22], we agree with the fourth and fifth hypotheses. Although some of the studies presented in Textbox 2 have not provided sufficient evidence in support of any conclusions, we regard this fact as indirect evidence for the fourth hypothesis as well. Furthermore, people’s privacy concerns, as presented in Table 4, may be influenced by the following factors: (1) context and type of technology; (2) age; (3) health needs; (4) personal trust and the device’s usability; (5) trade-off among privacy, autonomy, assistance, safety, or independence; (6) health status; (7) region; (8) gender; (9) user roles; (10) sociocultural context; (11) emotion; (12) previous knowledge; (13) personality; and (14) potential use of personal data.

### Research Question 2: How Will the Methodologies Influence Participants’ Privacy Awareness With Regard to Health Monitoring Technologies From the Perspective of Social Psychology?

Even if most of the studies satisfied the fourth and fifth hypotheses, the researchers’ confirmation bias could have influenced the results and participants’ answers. More specifically, in reviews, such bias exists when researchers search for evidence that can support their own beliefs [80]. For the other 3 approaches (qualitative, quantitative, and multimethod), researchers’ confirmation bias could also impact their interaction with participants, such as raising inducible questions or providing insufficient information [81]. It has already been pointed out that most people lack the cognitive ability to calculate privacy risks and to make rational privacy decisions because of incomplete information, bounded rationality, and information asymmetries [49]. Therefore, the information provided to the interviewees might compel them to give answers that match researchers’ expectations. For multimethod studies that include several experiments, the design of the experiments will also guide participants to make different privacy decisions; for example, because several studies found that there is a trade-off among privacy, autonomy, assistance, safety, or independence, we believe that a privacy-related question in the first experiment might encourage the participants to be concerned more about privacy rather than autonomy in the experiments that follow.

Furthermore, social influence in groups should also be emphasized because participants’ privacy awareness might be influenced not only by the sociocultural context but also by the other participants; for example, a herd mentality can lead participants to converge on a consensus answer and make irrational privacy decisions [82].

On the one hand, researchers found that the wisdom of small groups of people tends to outstrip that of both individuals and a large group of people. According to one of the findings, when there are 4 groups, and the number of participants in a focus group is 5, although opinions within a group might converge, there are still diversities among the different groups, and researchers will be able to gather different views from these groups [82]. On the basis of these findings, we analyzed the experiment design of focus groups in the selected studies and found that most experiments lacked diversity in terms of participants’ backgrounds [35,50,55,63].

On the other hand, even if the diversity in terms of participants’ backgrounds is enhanced, the results of a group cannot always represent personal privacy attitudes because of group polarization [83], that is, it remains questionable whether the decisions made by these groups can represent the views of individuals in the group accurately. In all, we cannot ignore the need and significance with regard to clarifying the ultimate goal of gathering and analyzing privacy attitudes.

### Legal Concerns and Legal Frameworks

Legal norms, overall, refer to social norms that are enforced by a relatively strong degree of coercion [84]. However, few of the articles we identified can be categorized into the topic of legal norms straightforwardly. Therefore, instead of categorizing them into legal norms, we classified the articles describing legal issues into 2 subcategories: legal concerns and legal frameworks. More precisely, the articles reflecting legal concerns were not describing participants’ concerns about the existing legal frameworks; rather, they were describing participants’ attitudes toward technology adoption in light of their awareness of legal obligations; for example, Sánchez et al [21] presented the fact that even if participants were aware of municipalities’ legal obligations to provide health care services for older people, they preferred to buy anything they could afford or adopt welfare technologies. The study by Sánchez et al [61] also did not present any legal concerns directly gathered from participants, but it highlighted the importance of legal liability for different user roles (physicians, nurses, or relatives of the patients) during a visit to patients and regarded it as legal concern. As a matter of fact, among the studies we selected, there were only a few conducted for gathering people’s legal concerns regarding privacy with respect to health monitoring and assisted living technologies. However, people’s legal concerns regarding privacy and the problems in current legal frameworks might be considered one of the influential factors when it comes to adopting health monitoring technologies, which constitute one of the privacy barriers.

Regarding legal frameworks, we extracted regulations, laws, policies, directives, and rules from the findings (Textbox 3). These documents are not limited to assisted living technologies; rather, they cover legal aspects in health care in general; for instance, the legal challenges in the home care or health care environment include data privacy, data management, stakeholders’ interests, and informed consent [85].

It is worth mentioning that not all legal documents concerning health monitoring or assisted living technologies are exhaustively listed in Textbox 3 because different countries have different laws or rules regulating aspects of health care. Some of the articles pointed out the shortcomings in the existing frameworks. Among these articles, Ryu [91] revealed the fact of the absence of legal guidelines in the mobile health domain regarding privacy and confidentiality in more than half of the EU countries and the United States and suggested that mobile health should be included within the framework in different countries; Ambrosino et al [92] provided the conclusion that a full legal framework for telemedicine was still lacking in European countries; and Sánchez et al [61] stated that the standardization, research, and assessment of the legal aspects should be addressed in an international perspective. However, in this paper, we only focus on the impact of legal norms on privacy concerns.

**Legal frameworks.**

**Study and the laws, rules, regulations, directives, and policies mentioned**

- Sánchez et al [21]: Norwegian Municipal Health and Care Services Act of 2011 (ACT 24/06/2011 no. 30; act relating to municipal health and care services, and so on)
- Gerłowska et al [51]: European Parliament resolution of February 16, 2017, with recommendations to the European Commission on Civil Law Rules on Robotics (European Parliament, 2017)
- Garg et al [86]: Health Information Technology for Economic and Clinical Health Act, United States (2009); Health Insurance Portability and Accountability Act, United States (1996)
- Costa et al [87]: Article 8 of the Charter of Fundamental Rights of the European Union (2010); Article 16 of the Treaty on the Functioning of the European Union (consolidated version of the Treaty on the Functioning of the European Union, 2012); Portuguese data protection law; General Data Protection Regulation
- Jin et al [88]: Health Insurance Portability and Accountability Act, United States (1996)
- Garzo and Garay-Vitoria [89]: Regulation 2016/679 (also known as General Data Protection Regulation); Regulation 2017/745 on medical devices (2017); Regulation 536/2014 relating to clinical tests with medication for human use (2014); harmonized standard ISO 14155 related to good clinical practice (International Organization for Standardization, 2020)
- Ross et al [90]: General Data Protection Regulation; Health Insurance Portability and Accountability Act, United States (1996)

### Research Question 3: What Are the Legal Challenges Regarding People’s Privacy Attitudes and Concerns Today?

The articles included in the review show the absence of research on legal norms regarding privacy or people’s legal concerns with regard to assisted living technologies in the health monitoring environment; for example, informed consent for various scenarios in health care, different user roles (eg, device owners, bystanders [68], and technology developers), and different types of health monitoring technologies require researchers to pay more attention to the legal frameworks rather than merely point out that they are inadequate.

To this end, a few of the included studies have investigated patients’ informed consent requirements in a health monitoring environment. As stated by Demiris and Hensel [93], when patients or older adults approach the end of their lives, they have opportunities to become familiar with smart home applications and perhaps change their minds and consent to use them in light of their value. Patients with cognitive impairment [68] who are gradually losing their cognitive ability to make decisions might prefer to disclose more information in exchange for better medical help when giving informed consent. Thus, informed consent requirements need to be updated.

This also applies to privacy decision-making in health care; for instance, informed consent could be obtained through a shared decision-making framework [93]. Generally, informed consent includes data processing, such as storage, transmission, collection, erasure, and sharing. However, when it is applied to a specific field, more concrete explanations of the risks and benefits need to be provided. In shared decision-making, which requires the involvement of patients and clinicians, informed consent serves as a legal process used to promote patient autonomy and self-determination as well as legal rights [94]. While shared decision-making includes treatment decision-making, it can also include, for example, privacy decision-making because it allows people to discuss how confidential information can be used and shared [95,96].

As shared decision-making is a collaborative process and aims to help patients better understand problems and make rational decisions with support from clinicians, both patients’ and clinicians’ opinions need to be taken into consideration. To be compliant with patients’ privacy needs and the cognitive changes they may be experiencing, we believe that informed consent requirements need to be updated continually as well [97].

The aforementioned cases only serve as examples of applications of legal concerns. More scenarios and elements remain to be clarified, such as identifying direct and indirect stakeholders and their responsibilities and distinguishing the need for informed consent when there are more user roles to be considered (formal caregivers as well as informal caregivers such as friends or relatives) in the health monitoring environment. Some scholars have pointed out that informed consent is not always necessary if the disclosure of information is consistent with respect for underlying human dignity or individual autonomy, which is referred to as “reasonable expectations of privacy” [98,99]. They argue there are circumstances in which confidential information can be better protected, precluding the need to rely on implied consent. By shifting from implied consent to “reasonable expectations of privacy,” the pressure to classify cases as *implied consent* could be eased [100].

As researchers are currently focusing more on reasonable expectations of privacy with regard to the sharing of confidential health information, reasonable expectations of privacy for adopting health monitoring and assisted living technologies or privacy decision-making concerning these technologies can possibly be taken into account in data protection legislation as well. Although we agree that reasonable expectations of privacy can help reduce participants’ burden when giving consent, the scope of reasonable expectations of privacy still relies on social psychological factors, such as the quality of the physician-patient relationship [99]; for example, trust between physicians and patients will increase the level of reasonable expectations of privacy when patients are making decisions, such as whether to allow the health monitoring system to send alerts to the clinician staff under some circumstances.

### Research Question 4: What Should Be Taken Into Consideration in Subsequent Studies Related to Privacy Attitudes and Concerns in the Context of Social Psychology?

Vassli and Farshchian [23] state that one of the most cited reasons that the authors found that might influence participants’ adoption of assisted living technologies was that monitoring devices made them feel observed. This has inspired us to suggest experiments (refer to the following paragraphs) that should be conducted in future studies.

First, as far as we could find, the selected studies had not looked into the problem of the Hawthorne effect [101], which refers to a phenomenon in which people alter their behavior in response to being watched or monitored, that is, they might make an instantaneous modification in their behavior once they become aware that they are being observed. People behave differently even when looking into a mirror (rather than being watched by someone else) [102]. In this sense, installing monitoring devices might affect people’s behavior even if they have consented to the use of these technologies.

Holden [103] suggested in 2001 that the possible presence of a Hawthorne effect could lead to participants drawing conclusions subconsciously. Therefore, we cannot predict the influences wrought by the Hawthorne effect, while this remains of key importance because it will consequently impact user experience and influence their decision-making in real life. Although some participants in the studies by Vassli and Farshchian [23] and Biermann et al [25] tended to ignore the feeling of being observed, the Hawthorne effect can cause positive impacts as well; for example, in the study by Cristiano et al [63], even if participants had negative feelings of privacy intrusion when being monitored, this was not always the case because older adults stated that they felt secure when being monitored. This also reflected the trade-off between privacy concerns and security concerns. The researchers claimed in their paper that negative feelings of privacy intrusion could be overcome by providing older adults with appropriate information. In another study of clinical trials in dementia, researchers who were aware of the Hawthorne effect found that more intensive follow-ups would cause better cognitive functioning outcomes [104]. Another observation from the Norwegian University of Science and Technology Nord-Trøndelag Health Study [103] showed that participants surveyed by the project regularly over many years exhibited statistically better health states than those not surveyed, which might be attributed to the Hawthorne effect as well. In this way, the feeling of being observed can turn out to be a good thing, although some technology researchers try to hide the monitoring devices to reduce patients’ feeling of being observed.

In all, the Hawthorne effect is a complex phenomenon that can lead to unknown bias. There should be more experiments to compare patients’ or older adults’ behaviors when they are aware of being observed and their behaviors with hidden observation during such research.

Second, in the follow-up experiment design of observing participants’ privacy behaviors, researchers should keep an eye not only on the privacy paradox phenomenon but also on the stress of cognitive dissonance caused by the phenomenon. The privacy paradox reveals the fact that there are discrepancies between users’ self-declared privacy attitudes and their privacy behavior [105,106]. These discrepancies will cause cognitive dissonance, which appears when people hold conflicting beliefs, or their behaviors contradict their beliefs [107,108].

It is mentally stressful to cope with contradictory experiences or beliefs, and cognitive dissonance will make conditions for patients or older adults in health care settings even more stressful [109]. But there can also be positive effects if researchers use a patient’s or an older adult’s motivation to mitigate the dissonance to change their behaviors [105]. A few researchers also found that the contrast between privacy concerns and privacy-protecting behaviors is caused by privacy fatigue [106], referring to the reduced intention of privacy protection when faced with the increasing complexity of privacy settings or regulations. Because of this, some participants even became confused about the laws or regulations and lacked the ability to make appropriate decisions or give consent [107].

Third, there is a lack of longitudinal studies on privacy attitudes with regard to assisted living technologies. Even if some studies had adopted multimethod approaches, and participants had been invited to take part in several experiments, it is hard for us to identify their cognitive changes over time. Because of the problem we have outlined in research question 1, current experiments might even induce participants to give the answers we want. Thus, we need long-term studies to test patients’ cognitive changes over the technologies.

### Overview

**Figure 2:**
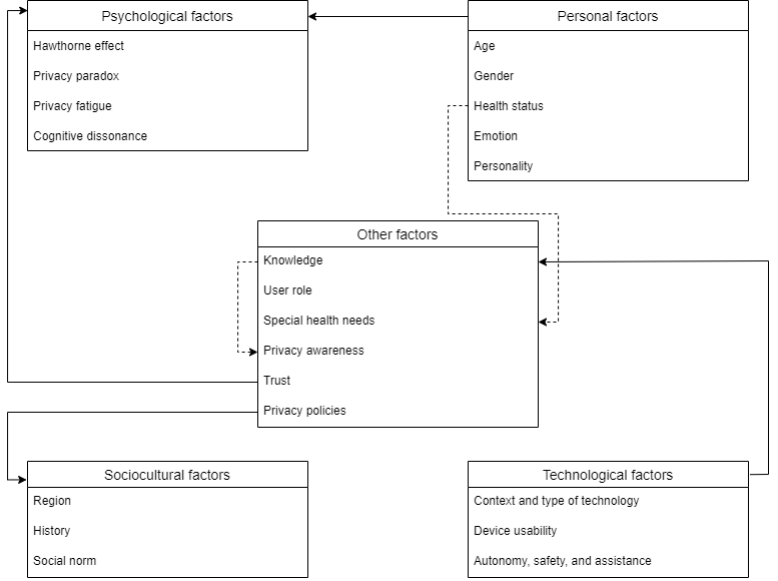
Influential factors of privacy concerns.

From the findings we extracted, we aimed to provide a comprehensive understanding of privacy barriers in health monitoring. We have explained the interaction of different factors, especially people’s privacy concerns and legal concerns, and pointed out the impact of social psychological factors on these factors. We suggest that to ensure people’s autonomy while protecting their privacy, the rules applied to them need to meet their demographic characteristics, health conditions, and health needs. Among the listed hypotheses and research questions, we tend to support the fourth hypothesis: people’s privacy concerns vary from person to person, and there is insufficient evidence to validate the importance of privacy barriers currently. As information asymmetries will also lead people to make different privacy decisions, we suppose that the more accurate and useful the information they provide, the more precise the decisions they will make. To intuitively present the influential factors we found in research question 1, we highlight the elements that should be considered and analyzed to measure a person’s privacy concerns (Figure 2). The categorization of the elements is flexible; for instance, both social trust and technical trust can affect privacy concerns (refer to the inner relations among the elements [solid lines] and subelements [dashed lines] plotted in Figure 2).

On the basis of these factors, we have determined that there are few studies investigating the privacy attitudes of other user roles with regard to these technologies. We found that, of the 66 included articles, only 1 (2%) [74] has conducted interviews with caregivers, while 2 (3%) [68,69] discuss the privacy concerns of bystanders. In addition, there are only a few studies that were not conducted within the specified geographic regions that collected technology researchers’ perceptions of ethical issues (privacy is one of the ethical issues interviewed) with regard to smart home technologies [108]. Although bystanders will not be the majority of the users of assisted living technologies, we insist that privacy concerns should be gathered from them too. In addition, clinicians’ and technical researchers’ views need to be explored because they can provide more information based on their professional background. Therefore, more studies are required to be conducted from the perspective of different user roles, enabling shared privacy decision-making among them.

This review also reveals the problems in current research, such as an insufficient number of participants recruited, a lack of diversity regarding focus groups, the confirmation bias of researchers during study design, and the fuzzy definitions of different concepts, and provides suggestions for some of the barriers especially from the perspective of social psychology, such as improving cognitive functioning by applying the Hawthorne effect or reducing cognitive inconsistency by using cognitive dissonance.

To help participants make more accurate and stable decisions, we suggest that more empirical studies should be conducted that observe participants’ behaviors and measure the distances between behaviors and attitudes. In combination with the self-perception theory [110], the participants are expected to observe themselves, notice the inconsistencies, and interpret their attitudes from their behaviors. In subsequent steps, researchers can also guide the participants appropriately based on the social learning theory [109], notify participants about the inconsistencies, and ask them to adjust their attitudes or behaviors to reduce the distances. On the basis of the newly gathered attitudes, participants’ preferences and behaviors in real life are expected to be predicted more precisely on the machine level, by using appropriate predicting algorithms.

### Study Limitations

Despite all the interesting findings, we acknowledge the limitations of the review. First, the scope of findings deviated somewhat from the search terms we set at the beginning. Because of the limited number of articles identified regarding legal concerns and legal frameworks, the legal frameworks and documents that we have listed are not exhaustive; therefore, we have not ventured in depth in this direction. Although we have classified the findings into subcategories of our creation, we cannot deny the fact that few studies are directly related to legal norms regarding privacy in health monitoring. Second, although some search terms were updated continually based on the new ideas we generated, to be compliant with the inclusion criteria (eg, the region specification), some important studies might have been excluded, although their findings may not be applicable and adaptable to the authors’ country of residence. However, we encourage future works to be carried out in other regions to obtain a more comprehensive overview of the problem. Last but not least, although we have tried to interpret the findings from the perspective of social psychology, the evidence we have presented is inconclusive, and they remain to be investigated in long-term studies.

### Conclusions

This scoping review has synthesized existing published research on privacy barriers with regard to the adoption of assisted living technologies. On the basis of the findings and main topics, the studies were classified into five categories: (1) privacy attitudes, (2) privacy concerns, (3) legal concerns, (4) legal frameworks, and (5) privacy barriers. Subsequently, we investigated the methodology and participant inclusion criteria. We have listed the factors that influence people’s privacy concerns and analyzed the social psychological influence of the experiments on people’s privacy awareness. Example legal challenges regarding privacy attitudes have been put forward, and the interaction between privacy factors and legal factors has been discussed. Future research might involve longitudinal studies on the privacy attitudes of different user roles and the informed consent obtained, with more psychological impacts such as the Hawthorne effect and confirmation bias carefully considered.

## Appendix

Multimedia Appendix 1 PRISMA (Preferred Reporting Items for Systematic Reviews and Meta-Analyses) checklist.

## Abbreviations

EU: European Union
PRISMA: Preferred Reporting Items for Systematic Reviews and Meta-Analyses
